# Unveiling the Long-Term Lung Consequences of Smoking and Tobacco Consumption: A Narrative Review

**DOI:** 10.7759/cureus.66415

**Published:** 2024-08-08

**Authors:** Srinivasulareddy Annareddy, Babaji Ghewade, Ulhas Jadhav, Pankaj Wagh, Souvik Sarkar

**Affiliations:** 1 Respiratory Medicine, Jawaharlal Nehru Medical College, Datta Meghe Institute of Higher Education and Research, Wardha, IND

**Keywords:** pulmonology, alcohol, peptic ulcer, hyperlipedemia, asthma, copd, lung cancer, smoking

## Abstract

Smoking and tobacco use present significant public health challenges due to their association with high morbidity and mortality rates worldwide. Despite reductions in smoking rates in many developed countries, global tobacco consumption remains high, especially in developing regions. This review examines the chronic effects of smoking on the respiratory system, detailing the pathological changes in the lungs and the resultant respiratory illnesses such as chronic obstructive pulmonary disease and lung cancer. Additionally, the review explores the impact of smoking on other body systems, including cardiovascular, immune, gastrointestinal, nervous, and reproductive systems. The extensive health implications of smoking emphasize the need for comprehensive public health interventions to reduce tobacco use and mitigate its adverse effects on health.

## Introduction and background

Tobacco use and smoking are some of the paramount public health challenges in the current society, which entails high morbidity and mortality rates. While the culture of smoking has been reduced in many developed countries, the overall rate of tobacco consumption across the globe remains drastically high, especially in developing countries [1]. It is established that more than 3 billion people in the whole world consume tobacco products, and around 8 million of the population perish annually from diseases related to tobacco consumption. The studies show that smoking also affects health in many other ways apart from the already discerned cardiovascular and oncological disorders, with the respiratory system carrying the bulk of the burden. This review seeks to establish the chronic effects of smoking and tobacco use with a focus on the pathological alteration in the lungs and types of respiratory illnesses that result from the impact of cigarettes [2,3].

The respiratory system is particularly susceptible to the adverse effects of using tobacco as it receives a direct impact from inhaled toxic materials. The major composition of tobacco smoke includes more than 7000 chemicals, out of which more than 100 are carcinogenic and 300 are irritants [4]. These substances initiate a series of pathological processes, such as long-term inflammation, oxidative stress, alterations in immune functions, and, as a result, morphological and functional deterioration of the lungs. Smoking’s severe and even irreversible negative consequences for the respiratory system can be evidenced by chronic obstructive pulmonary disease (COPD) development, lung cancer, and other pulmonary diseases. Over the past 10 years, with advancing analytical technology, significant amounts of new data have been published, increasing our understanding of levels of carcinogens in tobacco products [5]. Table 1 shows a few important chemicals from each carcinogenic compound found in tobacco smoke.

**Table 1:** Carcinogenic compounds found in tobacco smoke [5]

Class	Compound names
Polycyclic aromatic hydrocarbons	Naphthalene, benz[a]anthracene, benzo[c]phenanthrene, chrysene, 5-methylchrysene, benz[j]aceanthrylene, benzo[b]fluoranthene, benzo[j]fluoranthene, cyclopenta[cd]pyrene, indeno[1,2,3-cd]pyrene, dibenz[a,h]anthracene, benzo[a]pyrene, dibenzo[a,h]pyrene, dibenzo[a,i]pyrene, dibenzo[a,l]pyrene
Alkenes	1,3-butadiene, isoprene, styrene
Monocyclic aromatic hydrocarbons	Benzene, ethylbenzene, cumene
Aromatic amines	Aniline, ortho-toluidine, 2,6-dimethylaniline, 4-aminobiphenyl
Cyclic N-nitrosamines	N-nitrosopyrrolidine, N′-nitrosonornicotine, N′-nitrosonornicotine, N-nitrosomorpholine
Ethers	Ethylene oxide, methyleugenol, furan, benzofuran
Aldehydes	Formaldehyde, acetaldehyde, acrolein, crotonaldehyde

COPD is one of the major killers and a significant source of morbidity, and it mostly results from long-term smoking. It is a chronic disease that carries features of persistent cough and other respiratory signs, as well as limitations of airflow because of distortions within the airways and the alveoli. Disease-related conditions like chronic bronchitis and emphysema develop after long-term usage of tobacco smoke, which leads to chronic inflammation, excessive secretion of mucus, and destruction of alveoli. Lack of continued smoking significantly reduces the rate of worsening of COPD and remains the best treatment in the management of this disease. However, the disease continues to progress even after stopping smoking [6].

Another deadly effect associated with smoking is lung cancer, which remains responsible for the highest mortality rates of cancer in the world. Smoking is also closely related to lung cancer; tobacco smoking causes genetic changes and the malignant transformation of lung epithelial cells in both small-cell lung cancer (SCLC) and non-small-cell lung cancer (NSCLC). Also, smoking causes increased vulnerability to respiratory infections, increases the severity of asthma, and is associated with several Interstitial lung diseases. In the following review, these conditions will be elaborated on in detail, and this explains why there is a need for continued public health promotion campaigns against smoking and its impact on the health of the lungs [7].

## Review

Impact on various body systems

Respiratory System

Smoking has profound effects on the respiratory system. The inhalation of tobacco smoke introduces a plethora of harmful chemicals, including carcinogens, into the lungs, causing direct damage to the respiratory tissues. Chronic obstructive pulmonary disease, which encompasses chronic bronchial and emphysema, is caused by smoking. Chronic bronchitis is characterized by chronic cough and mucus production. In contrast, in emphysema, the alveoli are destroyed, the surface area that is available for gas exchange is reduced, and there is massive airflow limitation.

COPD is significantly linked to smoking, with never-smokers showing milder symptoms and lower risks compared to former and current smokers. A study by Nielsen et al. examined the effects of smoking on COPD outcomes using Danish health registries [8]. They used Poisson and Cox regression analyses to evaluate the exacerbation and mortality risks over 12 months for 49,826 COPD patients aged 40 and above who were diagnosed between 2008 and 2017. In comparison to former and current smokers, the study indicated that never-smokers with COPD had lower dyspnea levels, milder impairment of lung function, and lower risks of severe exacerbations and mortality. The risk of fatalities and severe exacerbations was highest among current smokers, highlighting the negative effects of smoking on COPD outcomes [8].

Madison et al. conducted research that indicates that extracellular vesicles (EVs) are involved in the pathophysiology of lung diseases like COPD [9]. Although EVs may contribute to COPD, there is no known connection between EV exposure and cigarette smoke (CS), which is the main risk factor for COPD. The study shows that exposure to CS can result in the formation of distinct EVs that contain matrix metalloproteinase (MMP-12) and neutrophil elastase (NE), both of which are essential for the development of emphysematous disease. The combination of NE and MMP-12-expressing EVs can cause lung damage and may even act as a predictor of the onset of COPD [9].

Moreover, socioeconomic and environmental factors beyond smoking play a crucial role in predicting and stratifying COPD risk, with a Socioeconomic and Environmental Risk Score (SERS) outperforming smoking status in predicting COPD development [10]. Additionally, psychiatric comorbidities, such as depression, are associated with continued smoking in COPD patients, emphasizing the importance of addressing mental health alongside smoking cessation for effective COPD management [11]. In a study conducted by Vicol et al., 52 patients with COPD were included [12]. The participants were split into three groups according to their smoking status: non-smokers, smokers, and ex-smokers. The study found that smokers and ex-smokers had higher levels of low-density lipoprotein cholesterol, lower levels of plasma high-density lipoproteins, and higher serum triglyceride concentrations. Additionally, smokers' low serum uric acid values may be a sign of chemical oxidative stress caused by tobacco smoke, which could disrupt lipid metabolism [12]

Lung cancer is directly associated with tobacco use, with estimates suggesting that smoking accounts for a number of lung carcinomas among males and females [13,14]. The interdependence between smoking and contact with other environmental factors, for example, radon progeny at the bifurcation of bronchi, increases the chances of lung cancer in smokers [15]. Smoke cigars contain several mutagens and carcinogens that cause changes in the genes of smokers, making them prone to developing lung cancer [14-16]. A person’s genetics determine his/her smoking activity, metabolism of carcinogens, DNA repair capabilities, and cellular signaling, among other factors that lead to lung cancer. Smoking cessation results in the improvement of the survival rate of lung cancer patients, including those who have undergone curative resection, because quitting this unhealthy habit effectively lowers respiratory cancer mortality [16].

The National Lung Screening Trial (NLST) was supported by the National Cancer Institute, comparing low-dose computed tomography (LDCT) with chest X-ray in lung cancer screening intervention in high-risk asymptomatic populations, mainly current smokers within the age range 55-74. The study revealed that LDCT decreased lung cancer mortality by 20% if compared to chest X-rays; that may be explained by the fact that early diagnosis is significant for high-risk smokers [17]. Brennan et al. undertook a study in the Journal of the National Cancer Institute through data from the International Lung Cancer Consortium [18]. Some of the questions that were studied were the genetic-environmental interactions in relation to lung cancer among smokers. Individual genetic factors were identified to have a very high influence on the probability of contracting lung cancer in smokers, in line with the interaction effect that the researchers established between genetic factors and tobacco [18].

As shown in a study conducted in 2008 by Bagaitkar et al., the subject of the research was the effect on the severity of respiratory infections due to smoking [19]. When comparing smokers to non-smokers, based on a group of patients with respiratory infections, the smokers contracted severe infections such as pneumonia and bronchitis than the non-smokers. This highlighted how smoking affects the mucociliary clearance mechanisms and lessens the body's ability to respond to infections and illnesses of the respiratory tract [19]. A systematic review by Huttunen et al. aimed to review the impact of second-hand smoke on respiratory tract infections among children [20]. The research indicated that children exposed to second-hand smoke had 1.5 times the risk of getting lower respiratory tract infections, ear infections, and lower respiratory tract infections. This means the different studies brought out the fact that eliminating or limiting second-hand smoking might actually lower the frequency of respiratory infections among young people [20].

In 2012, Polosa and Thomson conducted a study on the impact of smoking on asthma control in patients with the disease in adults [21]. The researchers also proved that current smokers had poorer control of their asthma with increased symptoms and severity than non-smokers. They also discovered that while former smokers did not smoke presently, they still exhibited a higher rate of asthmatic symptoms than non-smokers. Altogether, the study carried out proved that smoking cessation, indeed, has a positive impact on asthma outcomes. However, the benefits received were observed to be higher in cases when smoking cessation occurred at earlier stages [21].

Cardiovascular System

An important study that tried to investigate smoking habits and hypertension attempts was conducted by Thuy et al., a cross-sectional for former and current smokers; the investigators extracted data from the National Health and Nutrition Examination Survey and reported that both smoked groups had increased systolic and diastolic blood pressure, p < 0.001 [22]. The study acknowledged the fact that smoking causes endothelial dysfunction and oxidative stress, thus increasing arterial stiffness and hypertension. One of the most prominent works that analyzed smoking and coronary heart disease is the work of Whincup et al. [23]. This prospective cohort study included 20,000 patients with a follow-up of 10 years. It noted that those who had a smoking habit were two times more likely to develop coronary heart disease (CHD) as compared to non-smoking patients. The study also observed that the risk persisted even in the patients who had quit smoking, though the risk reduced with the period of cessation [22,23].

Hyperlipidemia and smoking are significant risk factors for atherosclerotic cardiovascular disease. Studies have shown that individuals exposed to second-hand smoke have a higher prevalence of dyslipidemia, especially hypertriglyceridemia, and high low-density lipoprotein cholesterol (LDL-C) levels [24]. Furthermore, in familial hypercholesterolemia pedigrees, smokers exhibit altered serum lipid profiles, with decreased apoA1 and increased apoB levels, indicating a higher risk of atherosclerotic cardiovascular disease [25]. Additionally, exposure to environmental tobacco smoke, along with high-perceived work stress, has been linked to hyperlipidemia, particularly in non-obese and non-smoking individuals, emphasizing the importance of reducing E-26 transformation-specific (ETS) exposure and workplace stress to prevent hyperlipidemia. Moreover, in hypertensive individuals, smoking is significantly associated with dyslipidemia and oxidative stress, with serum lipid levels showing a positive correlation with previous years of smoking [24]. These findings underscore the detrimental impact of smoking on lipid profiles and cardiovascular health.

Immune System

The relationship between smoking and reduced immune response has been reviewed and evidenced in prior research. A cross-sectional study has shown that tobacco smoke has toxic chemicals that impact immunity in both the innate and adaptive immune systems, resulting in changes in measures of immune cells, counts, cytokines, and functions [26,27]. Some research completed on active smoking in patients with head and neck squamous cell carcinomas (HNSCC) indicates that the immune system is reduced, with the lowest cytotoxic T cells and reduced immune signaling pathways to cancer [28]. In addition, cigarette smoke suppresses the ability of immune activation and effector cytokines, which subsequently reduces the immune response; smokeless tobacco extracts, on the other hand, have very little impact on the immune system [29]. Also, smoking and COPD have been found to alter cytokine responses in monocytes by diminishing their ability to produce cytokines in response to pathogens; this may offer an understanding of the high risk of bacterial infections in these patients [30].

Gastrointestinal System

Roshini et al. conducted a study on 100 patients to look into the effects of alcohol and smoking on peptic ulcer disease [31]. Smoking has been found to be a major contributing cause to the development of peptic ulcer disease, both in terms of its causation and its complications. However, in the patient population under investigation, alcohol was discovered to be an aggravating cofactor rather than the main cause of peptic ulcers. Tobacco smoking is directly related to the occurrence as well as the progression of peptic ulcer disease (PUD) [31]. Mendelian randomization analyses also provide evidence for the causality between smoking initiation and BMI, which exposes a relationship with gastric and duodenal ulcer risks [32]. Also, the survey-based study is characterized by a very significant relationship between current cigarette smoking and ulcer perforation, especially among the age group of more than 25 years [33]. In addition, a meta-analysis indicates that smoking is linked with PUD risk, and the latter accounts for most of smoking's impact on gastric cancer, especially among males, regarding the mechanism of stomach mucosal harm [34]. Mechanistic investigations also show that smoking has direct toxic effects on the cells lining the, as well as negatively affecting the mucosal immune system, which, in addition to verifying smoking's detrimental impact on ulcer development, can also be seen as a confirmation of the negative effects of tobacco for the gastrointestinal tract [35].

In order to learn more about the link between cigarette smoking and the risk of gastrointestinal cancer, the author, Hans Scherübl, reviewed and analyzed the body of prior research [36]. Hans Scherübl's research highlights the causal relationship between tobacco smoking and a number of gastrointestinal cancers, including anal, esophageal, gastric, pancreatic, biliary, hepatocellular, and colorectal cancers. It is important to address multiple risk factors because smoking increases the risk of gastrointestinal cancer when combined with other factors like alcohol use, excess body weight, diabetes, or chronic infections [36]. A systematic review was carried out by McMenamin et al. to look into the impact of alcohol and smoking on the prognosis of patients with gastrointestinal (GI) cancer [37]. Smokers who continued to smoke, especially those who smoked heavily, typically had a worse prognosis than those who never smoked for a variety of gastrointestinal (GI) cancers. The study indicates that programs encouraging patients with GI cancer to stop smoking may potentially improve their prognosis, but more research is needed to confirm the efficacy of the programs [37].

Nervous System

According to a study conducted by Hahad et al., smoking is a significant risk factor for neuropsychiatric diseases like dementia, schizophrenia, depression, anxiety disorders, and suicidal behavior, in addition to being a major risk factor for non-communicable diseases like cancer and cardiovascular disease [38]. A review of epidemiological studies was conducted to gather evidence on the relationship between smoking and these conditions. It has been realized that the health of non-smokers exposed to cigarette smoking is also at risk since it causes stroke and impairs the brain while having the reverse effect on Parkinson's disease [38]. Wang et al., in their study, included 69 healthy controls with and without a smoking history, as well as 129 patients with Parkinson's disease (PD), and recruited participants from the cohort [39]. It was discovered that the degeneration of dopaminergic neurons in PD causes a significant decrease in dopamine levels and dopaminergic afferent (DAT). Nicotine may function as a stimulant to block striatal DAT, thereby elevating dopamine levels in the synaptic gap. The inverse relationship between smoking and PD risk may be clarified by the inverse change in dopamine levels between nicotine addiction and Parkinson's disease [40]. Also, nicotine dependence raises age-related cortical factors, thinning, and cognitive loss, and smoking is a key risk factor for dementia. This is the reason why smoking cessation is effectively emphasized as an important component in dementia strategies [41]. In total, the findings presented demonstrate that smoking exerts negative effects not only on the global health status but also on neuro-, transmission-, and cognition-related processes in the human brain, which amplifies the necessity of further development of effective means and actions to limit the dangers connected with tobacco agents and products [42].

Reproductive System

Smoking poses significant harm to the reproductive systems of both male and female individuals. Studies show that smoking also affects sperm, hormones, hormonal axes, and antioxidants in semen, which results in abnormal semen quality in male reproductive health [43]. Females are affected by smoking in that it causes gamete mutagenesis, early decline of reproductive function, ectopic pregnancy, spontaneous abortion, early menopause, and the need for assisted reproductive technology for conception [44]. Previous investigations that utilized mice treated with cigarette smoke condensate (CSC) found that overall conception rate, sperm quality, oocyte quality, and in vitro fertilization rate were significantly or numerically reduced, indicating that smoking lowers fecundity and offspring number [45].

Co-relation With Tuberculosis and Pneumonia

Smoking has been extensively studied in relation to tuberculosis (TB), with evidence pointing to its significant impact on TB infection, disease progression, severity, and mortality [46]. Studies have shown that both active and passive smoking are independent risk factors for TB infection and reactivation, with smokers having higher rates of latent TB infection compared to non-smokers. Furthermore, smoking cessation interventions, such as behavioral change communication (BCC) and BCC plus bupropion, have been proven effective in improving TB treatment outcomes and promoting smoking abstinence among TB patients, highlighting the importance of incorporating smoking cessation programs into TB control strategies [47]. Additionally, meta-analyses have demonstrated that smoking increases the risk of TB recurrence, emphasizing the need for interventions to reduce smoking rates to achieve TB control goals by 2030 [48]. The study by Pourali et al. concluded that smoking significantly increases the risk of tuberculosis recurrence. The odds ratio for TB recurrence in smokers was found to be 2.10 times higher compared to non-smokers, indicating a strong association between smoking and the likelihood of experiencing TB again [49].

Smoking is significantly associated with an increased risk of contracting and dying from pneumonia, as highlighted in various research studies. Current smokers have a higher hazard ratio of contracting pneumonia and experiencing mortality compared to non-smokers, with risks increasing in a dose-response manner with the number of pack years smoked [50]. Additionally, smoking status at the time of hospitalization with pneumonia is linked to a higher risk of readmission with recurrent pneumonia within a year of discharge, with current smokers and people who quit smoking showing elevated risks compared to never-smokers [51]. Furthermore, exposure to cigarette smoke weakens respiratory defense mechanisms, making individuals more susceptible to various respiratory infections, including pneumonia, emphasizing the critical importance of smoking cessation for both primary prevention and reducing pneumonia-related morbidity and mortality [52].

Involvement of Various Other Organs

Smoking plays a significant role in the development and prognosis of cancers in the oral cavity. Research indicates that smoking is a primary risk factor for oral squamous cell carcinoma (OSCC), with a strong association between smoking and alcohol use in OSCC of different oral cavity sites, particularly on the floor of the mouth (FOM) [53]. Furthermore, a study by Eloranta et al. found that current and former smokers had a higher risk of mortality from oral cavity and pharyngeal cancer (OPC) compared to non-smokers, emphasizing the impact of smoking on cancer-specific survival outcomes [54]. Additionally, historical studies have shown that smoking, along with alcohol consumption, accounts for a significant proportion of oral cavity and pharyngeal cancers [55,56]. 

Smoking plays a significant role in the development of cancers in the larynx and pharynx. Studies have shown that smoking is a leading risk factor for pharynx and larynx cancers, contributing to a substantial portion of the disease burden [57]. Specifically, bidi smoking has been identified as a stronger risk factor compared to cigarette smoking for hypopharyngeal and supraglottis cancers in India, emphasizing the detrimental impact of tobacco products on these specific cancer types. Additionally, the association between smoking and alcohol consumption with laryngeal cancer has been explored, revealing a significant correlation between smoking status and the presence of laryngopharyngeal reflux (LPR) in patients diagnosed with laryngeal cancer, highlighting the interconnectedness of smoking, alcohol use, and the development of these cancers [58].

Smoking is strongly linked to an increased risk of cancers of the esophagus and pancreas. Research has shown that smoking is associated with a higher risk of esophageal cancer, with a hazard ratio (HR) of 3.09 for the development of this type of cancer [59]. Additionally, tobacco smoking is a known risk factor for pancreatic cancer, with cigarette smokers having a higher likelihood of developing this disease compared to nonsmokers. The mechanisms through which smoking contributes to pancreatic cancer are still not fully understood. Still, it is established that smoking is a major risk factor for chronic pancreatitis and pancreatic cancer [60].

Discussion

The changes that smoking causes are numerous and affect every body system, which has confirmed that smoking is a severe problem that negatively influences people's health. In the respiratory system, smoke is a chief culprit in COPD and is responsible for lung cancer as well [8-12]. Research shows that smoking and ex-smoking COPD patients have worse symptoms and higher rates of exacerbations and mortality compared to smokers. More importantly, it shows that lung damage resulting from smoking is conditioned by factors of a socioeconomic and environmental nature, thus suggesting the application of complex population policies in response to the problem, in addition to smoking control measures [13-16].

Smoking severely affects cardiovascular health by causing hypertension, endothelial dysfunction, and an increase in lipid levels in the blood, which raises the chances of the development of coronary heart disease and stroke. Smoking has also been implicated in affecting lipid profiles, and hence, smoking poses a multiple risk factor for atherosclerotic cardiovascular diseases. Also, passive smoking leads to a significant cardiovascular danger; therefore, people should avoid smoking and have minimal exposure to second-hand smoke [23-25].

Smoking has adverse effects on hematology/immunologic, gastrointestinal, neurologic/psychiatric, and reproductive systems. Smoking weakens a person's general immune system, which can result in increased vulnerability to certain diseases and cancers. It predisposes patients to diseases affecting the gastrointestinal tract, including peptic ulcer disease and various cancers affecting the gut [29-35]. Smoking is connected with neuropsychiatric disorders and recurrent brain impairments in cognition and neuromodulation in general; on the level of the reproductive system, it is associated with provoked decreased fertility rates and adverse effects on pregnancy. The evidence highlights the significant impact of smoking on TB, pneumonia, and various cancers. Smoking increases the risk of TB infection, reactivation, and recurrence, underscoring the need for integrating smoking cessation programs into TB control strategies. For pneumonia, smoking raises the risk of infection, severity, and mortality, emphasizing the importance of quitting smoking for prevention and better outcomes. Smoking is a major risk factor for cancers of the oral cavity, pharynx, larynx, esophagus, and pancreas, with strong links to higher incidence and mortality [47-60]. These and all the above findings together call for strong smoking cessation and other effective public health initiatives to remediate the broad range of adverse health impacts that accompany smoking.

## Conclusions

Regarding the various body systems, smoking has been associated with respiratory diseases like chronic obstructive pulmonary disease and lung cancer, cardiovascular diseases such as hypertension and coronary heart disease, as well as immunological, gastrointestinal, neurological, and reproductive diseases. The multitude of findings underlines the necessity of complex preventive measures spanning population-based smoking control, decreasing exposure to second-hand smoke, and tackling the multiple factors of social deprivation. Public health prevention efforts targeting cigarette smoking are needed to reduce the number of people affected by smoking-related diseases and enhance the health of the population.
